# Influence of Polymer Matrices on the Tensile and Impact Properties of Long Fiber-Reinforced Thermoplastic Composites

**DOI:** 10.3390/polym15020408

**Published:** 2023-01-12

**Authors:** Lijuan Jiang, Yinzhi Zhou, Fengnian Jin, Zhenhua Hou

**Affiliations:** 1 International Institute for Urban Systems Engineering, Southeast University, Nanjing 210096, China; 2 College of National Defense Engineering, Army Engineering University of PLA, Nanjing 210007, China; 3 Jiangxi Xinda Hangke New Materials Technology Co., Ltd., Nanchang 330096, China; 4 International Institute of Materials Innovation, Nanchang University, Nanchang 330031, China

**Keywords:** fibers, thermoplastic, tensile properties, impact properties, fracture modes

## Abstract

To investigate the influence of polymer matrices on the tensile and impact properties of long fiber-reinforced thermoplastic (LFT) composites, composites of long basalt fiber-reinforced thermoplastic were developed in this work. Two types of polyethylene, namely 8008 and 100S, and two types of polyethylene, namely C4220 and K8303, were chosen as the matrices. The fiber volume fractions were set as 2.8%, 5.9%, 8.1%, and 10.6%. The melt flow index (MFI), crystallinity, tensile properties, impact strength, and fracture morphology of the neat polymers and the corresponding composites were tested. The composites of 8008 showed the highest tensile strength since neat 8008 showed a much higher MFI value and crystallinity. The composites of 8008 and K8303 showed a much higher tensile modulus since the neat thermoplastic showed a higher tensile modulus than the other two composites. The polymer toughness was the factor that determined whether the polymer could be toughened by fibers. Moreover, the interfacial shear strength was calculated and compared with the matrix shear strength, based on which fracture modes of the LFT were predicted. Effective methods were proposed for further improvement of the mechanical properties. The results of this paper were essential for attaining the anticipated properties when designing LFT composites.

## 1. Introduction

Recently, thermoplastic polymers have experienced tremendous application growth in pipeline engineering owing to their low cost, excellent resistance to chemical agents, and increased recyclability [1,2]. However, their relatively poor mechanical properties limit the development of pipelines with a large diameter and high bearing capacity, which requires higher tensile strength to resist internal pressure, a higher tensile modulus to improve the ring stiffness, and a higher impact strength to resist dynamic loads during construction and service.

One of the most important methods to improve the mechanical properties of thermoplastic was to reinforce the thermoplastic with a second-phase material, such as continuous fibers, inorganic particles, and discontinuous fibers. However, due to the high viscosity of thermoplastic, continuous fibers cannot easily be impregnated evenly in the resin. Adding an organic solvent to the molten resin is thought to be an effective way to reduce its viscosity; however, it weakens the interface between the fiber and the resin. Particularly, if the solvent is not sufficiently volatilized, it will leave pores inside the composites, which will further reduce the performance of the composites. Inorganic particles, such as CaCO_3_ and quartz powder, are also usually adopted in reinforced thermoplastic and certain enhancements have been achieved. However, as the inorganic particles are usually polar, their interface bonding with non-polar resin is poor. In addition, the distribution of inorganic particles destroys the continuity of the resin, which will reduce the performance of the composite materials, especially the impact performance.

Short fiber-reinforced thermoplastic (SFRT) composites have drawn much academic and industrial attention due to their high performance, easy preparation, good designability, and low cost [3,4,5]. The extrusion-injection process is the most widely applied technology in SFRT manufacturing. However, in this process, fibers are usually shortened owing to the strong shear force of the screw, which has proven to be inefficient in reinforced thermoplastic.

Long fibers are intensively used to reinforce thermoplastic polymers. Long fiber-reinforced thermoplastic (LFT) composites are prepared using a novel technology to manufacture discontinuous fiber-reinforced composites whose fiber lengths can be as long as 50 mm, and it has proved to achieve better tensile performance compared to standard SFRT [6,7,8,9]. However, it was found in the engineering that the enhancement efficiency varied for different resins even with the same LFT technology and the same fiber parameters. So, which kinds of thermoplastic polymers can be reinforced by long fibers? Which property of the matrix determines the final mechanical performance of LFT composites? It is of great importance to investigate the influence of polymer matrices on the tensile and impact properties of LFT composites.

PE and PP were the most used in short and long fiber composites, due to their lower processing temperatures, better recyclability, lower density, and cost compared to other thermoplastics [10]. Several studies have focused on matrix polymers. Shah [11] investigated the quasi-static crush performance of thermoplastic 3D-FRC, and the results were compared with the conventional thermoset 3D-FRC. The thermoplastic composite showed much better mechanics due to the higher fragmentation failure mechanisms, which absorbed more energy. Carvalho [12] conducted research on the influence of the matrix melt flow index (MFI) on wood particle-reinforced high-density polyethylene (HDPE) composites. The results indicated that MFI played an important role not only in the mechanical properties of the matrix itself but also in the wettability between the particles and the matrix. Gomes et al. [13,14] also reported that the higher the MFI, the poorer the tensile and impact strengths of the matrix, but the better the mixture and process conditions. Fu [9] reported that a lower-MFI matrix usually led to a strong interaction between the fibers and the matrix, resulting in a lower mean fiber length; therefore, the fiber-reinforcing efficiency of the composite decreased. Franciszczak [15] found that the matrix crystallinity affected the mechanical properties of short fiber-reinforced polypropylene (PP) composites. An increase in the PP matrix crystallinity had a significant positive influence on the tensile and flexural performance due to the improved stress transfer from the matrix to the fibers. Lu [16] investigated the differences between hemp fiber-reinforced recycled HDPE (rHDPE) and virgin HDPE (vHDPE) composites. The results showed that rHDPE was stronger than vHDPE and had better wettability of the hemp fibers, resulting in better mechanical properties of the composites.

However, opposite conclusions were obtained regarding the toughness of the composites reinforced with discontinuous fibers. Langer et al. [17,18] highlighted the positive influence of short fibers on the composite toughness owing to the introduction of fiber-related energy dissipation, which did not occur in the single component. However, Garcia-Gonzalez [19] reported quite the contrary conclusion, where a lack of energy dissipation by polymer plastic deformation caused by the addition of fibers played a decisive role in the composite toughness. Which kinds of energy dispassion mechanisms will occur and which mechanism will play the decisive role should be further investigated [20]. In addition, although shear failure was thought to be the dominant damage mode for neat HDPE and PP, the crack initiation mechanisms were different in the LFT composites [21,22,23].

Jiang [6] created a review of the latest research developments on LFT composites and summarized that the influence of the matrix concerns four factors: viscosity, crystallinity, yield strength, and ductility. However, these previous studies are far from sufficient because they were mainly limited to one or two parameters, and it is not clear which parameter dominates. In addition, the research results are mostly qualitative. The current study intends to provide a comprehensive understanding of the influence of polymer matrices on LFT composites. For this purpose, two types of HDPE and two types of PP with different physical and mechanical properties were applied to compare the performances of different long fiber matrix systems subjected to the same processing conditions. The MFI, crystallinity, tensile properties, and impact strength of the neat polymers and the corresponding composites were tested, and the microstructures were observed. In addition, the interfacial shear strength of the composites was also calculated, and the fracture modes were predicted. Effective methods are proposed for the design of LFT composites.

## 2. Materials and Methods

### 2.1. Raw Materials and Specimen Preparation

Two types of HDPE, namely DMDA8008 (denoted as 8008, Dushanzi Petrochemical Company, Karamay, China) and JHMGC100S (denoted as 100S, Jilin Petrochemical Company, Jilin, China), and two types of copolymer-polypropylene (cPP), namely C4220 and K8303 (both provided by Yanshan Petrochemical Company, Beijing, China), were used as matrices for composite preparation in this study. The basic physical and mechanical properties provided by the manufacturers are presented in Table 1. Basalt fiber (Jiangsu GMV Co., Ltd., Nanjing, China) was used as reinforcement. The single fiber diameter of the basalt fiber is 13 μm, the tensile strength is 2100 MPa, the tensile modulus is 87 GPa, and the break elongation is 2.5%. In this study, the fibers were cut to lengths of 8 mm by the LFT process, which in our previous studies showed great advantages over other fiber lengths. The fiber volume fractions were set as 2.8%, 5.9%, 8.1%, and 10.6%. The corresponding composites were denoted as C-8008-X, C-100S-X, C-C4220-X, and C-K8303-X, where “X” represented the fiber volume fraction.

**Figure 1:**
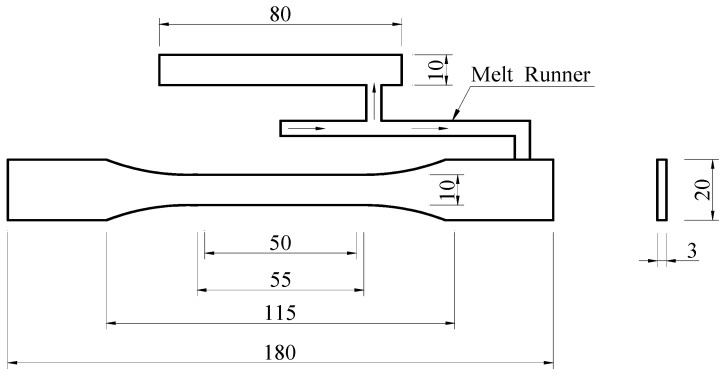
Specimen size and injection melt flow direction.

After the LFT process, the specimens were produced through an injection process according to Chinese standard GB/T1447. The specimen sizes and melt flow directions are shown in Figure 1. During injection, the temperature was set to 220 °C, and the injection molding pressure was set to 8−10 MPa. It should be noted that when the fiber volume fraction is increased, the injection molding pressure should also be increased until a small amount of matrix overflows on the edge of the molds.

### 2.2. Testing Setup and Data Processing

#### 2.2.1. Melt Flow Index (MFI)

The MFI is a characteristic of polymer viscosity. The MFI values of the LFT composites and neat polymers were measured using a fusion index instrument (TY5005, Tianyuan, Yangzhou, China) according to ISO 1133-1:2011. The test conditions were set with a temperature of 190 °C and a weight of 2.16 kg, and the mass of the outflow melt was weighed.

#### 2.2.2. Crystallinity

The crystallinity was measured using differential scanning calorimetry (DSC; STA449 F3, Netzsch, Bayerische, Germany) according to ISO 13779-3:2008. Before the tests, the samples were first ground into a powder, heated to 200 °C at a rate of 10 °C/min, held at this temperature for 5 min, and then cooled to room temperature at a rate of 10 °C/min to eliminate the previous thermal history. The specimens were then reheated to 400 °C at a rate of 10 °C/min. N2 was used as a protective atmosphere. Then, the crystallinity was calculated as follows:

(1) $$x_{c}=\frac{\Delta H_{m}}{\left(1-\lambda \right)\Delta H_{m}^{0}}\times 100\%$$

where $\Delta H_{m}$ was the actual melting entropy of the test specimens, $\Delta H_{m}^{0}$ was the theoretical melting entropy of the corresponding pure matrix when the crystallinity was 100%, and *λ* was the fiber mass fraction.

#### 2.2.3. Tensile Property

The tensile strength and modulus of the LFT and neat polymers were tested using a 10-kN universal testing machine (AG-Xplus, Shimadzu, Kyoto, Japan) according to Chinese standard GB/T1447. The tests were conducted in the displacement-control mode at a rate of 2 mm/min. In this study, the tensile loading direction was parallel to the melt flow direction. 

The tensile strength, σ (MPa), is obtained by Equation (2):

σ = F/(b·h)(2)

where F is the ultimate force before the SFRP failure in N; b is the width of the SFRP in mm; and h is the thickness of the SFRP in mm.

The tensile modulus of the elastic, E (GPa), is obtained by Equation (3) according to ASTM D7205:

E = ΔF/(b·h·ε)(3)

where ΔF is the difference in force between 20% and 50% of the ultimate force in the linear stage in N. ε is the difference in the strain corresponding to the two force points. The strain ε is obtained by strain gauges pasted on the surface of the SFRP.

Tests were conducted on at least five identical specimens for each sample type, and the average result was obtained as the final properties.

#### 2.2.4. Charpy Notched Impact Strength

The Charpy notched impact strength was measured using a pendulum impactor (PIT501J-TS, Wance, Shenzhen, China) according to ISO 179-1:2010. The specimens were 80 mm long, 10 mm wide, and 4 mm thick, and the “V” notch was 2 mm deep. The support span was set as 60 mm. The impact load was applied to the back of the “V” notch perpendicular to the melt flow direction. Ten identical specimens for each sample condition were tested, and the average was obtained.

#### 2.2.5. Microstructure Observation

The microstructures of the fracture surfaces were observed using a JSM-6510 scanning electron microscope (SEM; JEOL, Tokyo, Japan). Before observation, the fracture surfaces were cleaned with ethanol and vacuum dried. Then, they were sprayed with gold for 45 s using a diode-sputtering coater.

## 3. Results

### 3.1. MFI

The MFI values of the neat matrices and the corresponding LFT composites are listed in Table 2.

For the neat polymers, the 8008 showed the largest MFI value, whereas 100S showed the smallest MFI value. The MFI has been proven to be dependent on the polymer molecular and molar distribution [28]. Generally, the lower the molecular weight and the narrower the molecular weight distribution, the higher the MFI. Because 8008 had the minimum Mn and Mw values, as well as the minimum distribution coefficient D, it showed the largest MFI. Because 100S had the largest distribution coefficient, D, it showed the smallest MFI.

The addition of the fibers significantly reduced the MFI of the composites. For C-8008-10.6%, the MFI was reduced by 24.47%. For C−100S−10.6%, the MFI was reduced by 72.00%. For C−4220−10.6% and C−K8303−10.6%, no melt flew out under the given test conditions. Thus, the MFI values for these composites were not obtained. A decreased MFI indicated greater viscosity, poorer flow capability, and worse processing performance of the materials.

### 3.2. Crystallinity

The crystallinities of the neat matrices and the corresponding LFT composites are listed in Table 3.

The crystallinity of thermoplastics is primarily related to the regularity of the molecular chains of the polymer. The crystallinity of the neat polymers follows the order as follows: 8008 > 100S > K8303 > C4220. The incorporation of fibers disrupted the integrity of the molecular chain. Thus, the crystallinity degree was reduced by 9.2% for C−8008−2.8%, 6.5% for C−100S−2.8%, 1.7% for C−K8303−2.8%, and 7.5% for C−C4220−2.8%.

### 3.3. Tensile Properties

**Figure 2:**
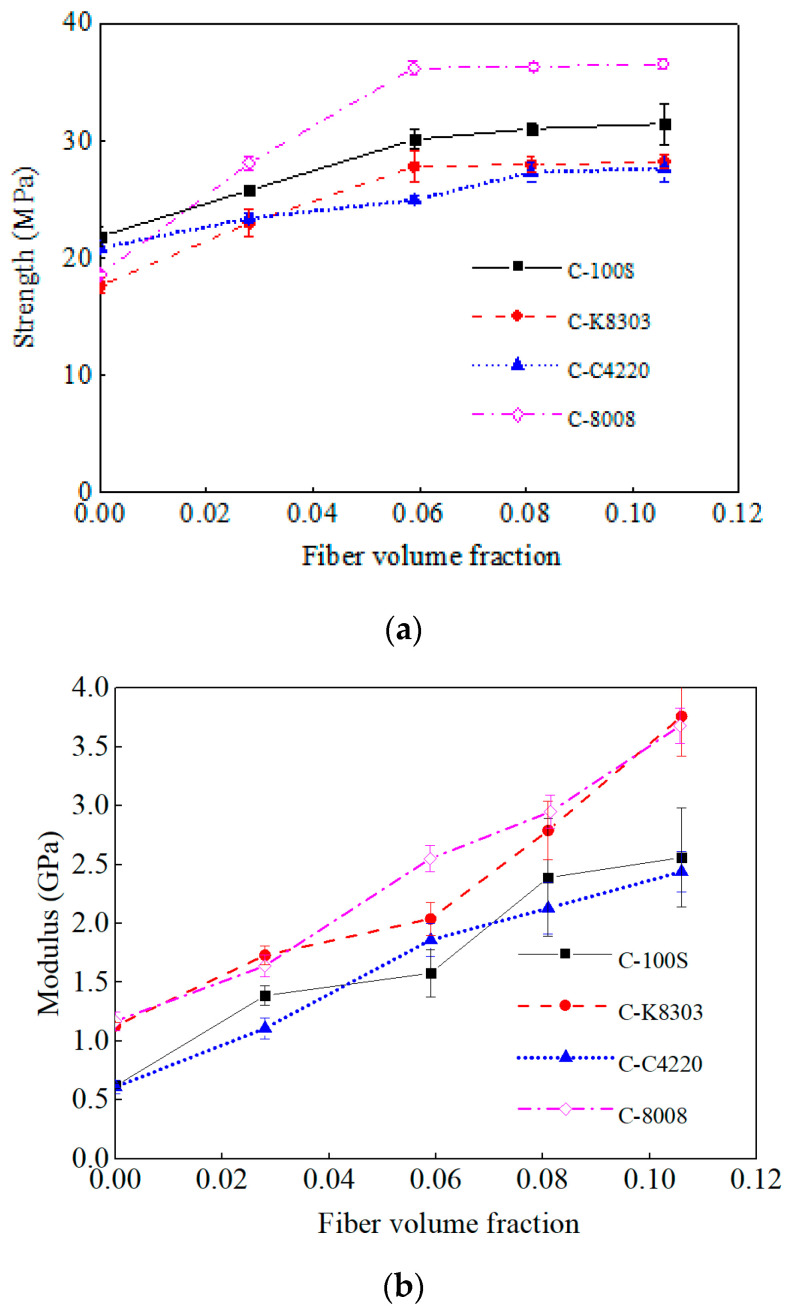
Tensile properties of the neat polymers and LFT composites. (**a**) tensile strength; (**b**) tensile modulus.

The tensile strength and modulus of the neat polymers and LFT composites are depicted in Figure 2.

The tensile strengths of the composites with different matrices showed similar trends: the tensile strength initially increased linearly with the increasing fiber volume fraction and then the strength increase slowed down and maintained an almost constant strength. Because fibers play the main load-bearing role in composites, thus increasing the fiber volume fraction, the tensile strength of the composites improved. However, as the fiber volume fraction further increased, fiber agglomerations occurred, particularly in the width and thickness direction, which usually acted as stress concentrators upon loading. This explained why the tensile strength of the LFT composites no longer increased with higher fiber fractions.

The tensile modulus for each LFT composite increased almost linearly with the increasing fiber volume fraction. There are two main reasons attributed to this. Firstly, the addition of the fibers increased the composites’ tensile strength. Secondly, the fibers restricted the movement of the polymer chain segments, thus the composite deformation decreased. 

### 3.4. Notched Impact Strength

**Figure 3:**
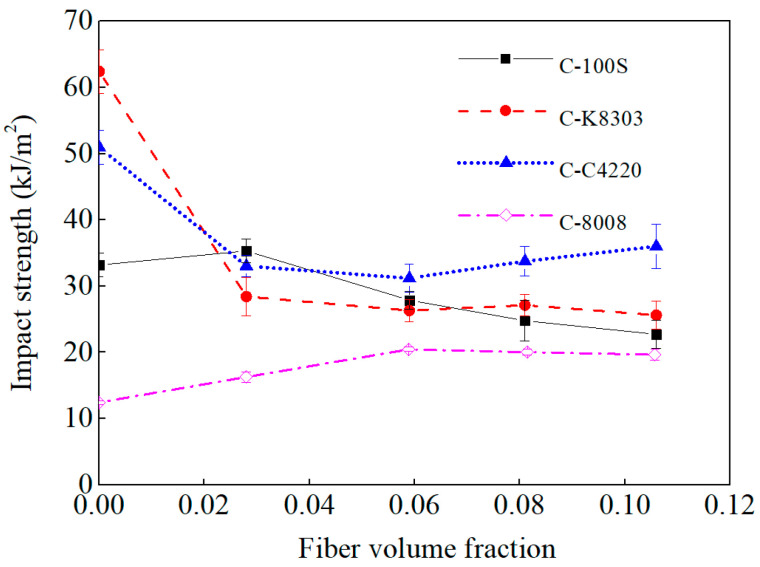
The notched impact strengths of the neat polymers and LFT composites.

The notched impact strengths of the neat polymers and the LFT composites are also shown in Figure 3. For the neat polymers, the notched impact strength followed the order of K8303 > C4220 > 100S > 8008. The larger notched impact strength of K8303 and C4220 can be attributed to the fact that there are ethylene segments in their molecular chain, as well as large amorphous areas, both of which can provide adequate toughness when subjected to impact loads. A similar situation prevails in 100S, which showed a typical double-peak structure in the molecular chain. The amorphous region in the 100S molecular chain is much larger than that of 8008, thus it showed a much higher impact strength than 8008.

With the incorporation of the fibers, the LFT composites’ notched impact strengths showed distinct responses. For C−8008, as the fiber volume fraction increased, the notched impact strength increased slowly initially and then remained almost constant. For C−100S, the notched impact strength increased first. For C−100S−2.8%, the notched impact strength increased by 6.4%. Then, the notched impact strength began to decrease monotonously with the increasing fiber volume fraction. For C-100S-10.6%, the notched impact strength decreased by 31.5%. For C−K8303, the notched impact strength drastically decreased at first. For C-K8303-2.8%, the strength decreased by 54%. Then, the notched impact strength remained almost constant, which was about 42% of the neat K8303. For C−C4220, the notched impact strength drastically decreased first and decreased by 35% for C−C4220−2.8%. Then, it increased slowly with the increasing fiber volume fraction.

## 4. Discussion

### 4.1. Effect of Polymer MFI

The MFI has been proven to be directly linked to the fiber dispersion efficiency in the polymer. Generally, the larger the MFI value, the better the fluidity and processability of the polymer, as well as a more uniform fiber distribution in the polymer [29,30]. Because 8008 showed the largest MFI value, the fibers can be more efficiently wetted by it and it can exhibit a higher load transference between the fibers and matrix. Thus, C−8008 showed much higher tensile strength than the other three composites with the same fiber volume fraction.

### 4.2. Effect of Polymer Crystallinity

As the LFT composites cooled down during the preparation, owing to the mismatch of the thermal expansion coefficients between the fibers and the matrix, the thermoplastic polymer oriented with the fiber under the action of thermal stress. Simultaneously, the fiber provided a nucleation surface for the thermoplastic resin [31]. Based on these two mechanisms, the nucleation barrier for crystallization was reduced, and the crystalline regions grew from the fiber surface. Thus, the interfacial shear strength increased. Moreover, with a higher crystallinity, the chain mobility of the polymer was constrained by the crystalline phase. Thus, the interface stress relaxation was reduced [32,33]. Therefore, a higher crystallinity usually indicated a higher stress transfer efficiency [34,35,36]. This also explained the much higher tensile strength of C−8008 compared to the other composites.

### 4.3. Effect of Polymer Tensile Properties

As can be seen from Figure 2a, although neat 8008 showed much lower tensile strength than the other three polymers, C−8008 showed the highest tensile strength among all the LFT composites with the same fiber volume fraction. It can be deduced that the polymer tensile strength was not the determining factor for the LFT composites’ tensile properties. Instead, a higher MFI value and higher crystallinity were more important in the LFT composites’ tensile strengths.

However, the tensile modulus of the LFT composites was obviously determined by the matrix tensile modulus, as neat 8008 and neat K8303 had a higher tensile modulus than C4220 and 100S and C−8008 and C−K8303 showed a much higher tensile modulus than C−C4220 and C−100S with the same fiber volume fraction. 

It also should be noted that the curves of the fiber volume fraction vs. the tensile modulus for C−100S, C−K8303, and C−C4220 deviated from a straight line. This was because the MFI values of 100S, K8303, and C4220 were very small. With an increased fiber volume fraction, fiber agglomerations easily occurred, resulting in inhomogeneous deformations. This resulted from the instability of the calculated modulus.

### 4.4. Effect of Polymer Notched Impact Strength

**Figure 4:**
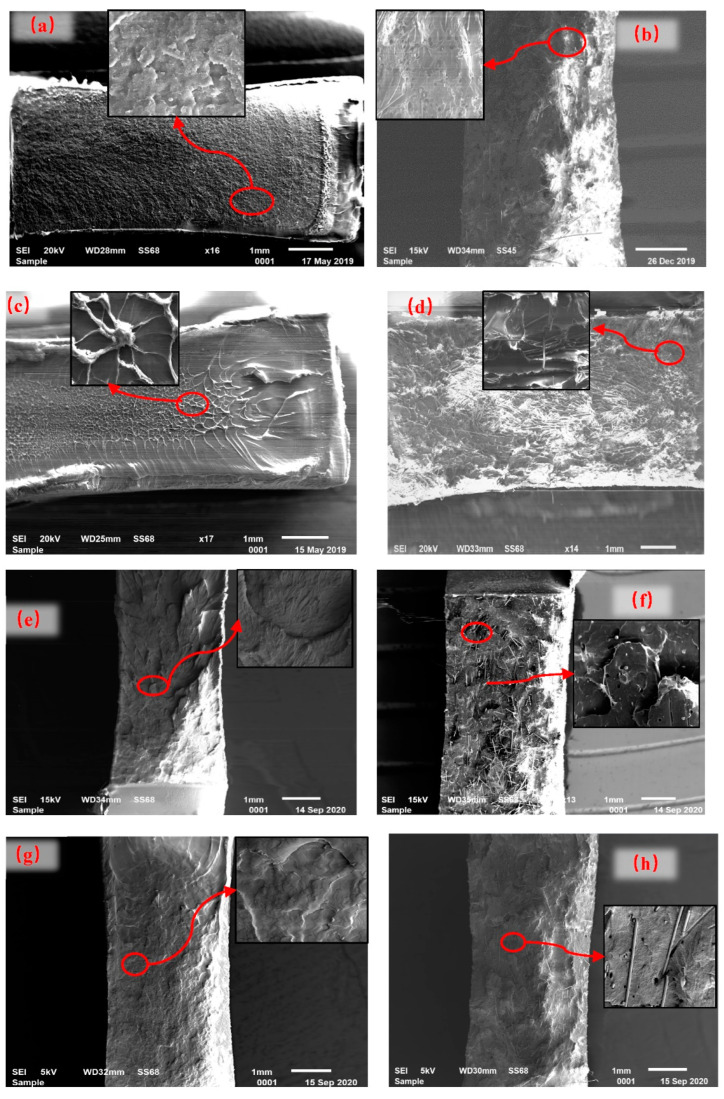
Fracture surfaces for the neat polymers and LFT composites. (**a**) 8008; (**b**) C−8008−2.8%; (**c**) 100S; (**d**) C−100S−2.8%; (**e**) K8303; (**f**) C−K8303−2.8%; (**g**) C4220; (**h**) C−C4220−2.8%.

To reveal the different mechanisms of the notched impact strength with the incorporation of fibers, SEM images of the fracture surfaces of the neat polymers and the corresponding LFT composites with a 2.8 vol.% of fibers are shown in Figure 4.

The fracture surface of neat 8008 (Figure 4a) was flat. As shown in the higher magnification, there were cleavage fractures, which can be visualized as a sudden burst of energy on the breakdown, indicating a relatively brittle fracture mode [37]. C−8008−2.8% (Figure 4b) exhibited a much more ductile morphology than neat 8008, with a rougher fracture surface. As shown in the higher magnification, large numbers of fibers were pulled out from the matrix, and due to the stress concentrated during the fiber pull-out process, plastic deformation appeared. Both of these two mechanisms consume a large amount of energy. Therefore, the notched impact strength of C−8008−2.8% increased. As the fiber volume fraction further increased, more fibers were pulled out during the impact load. Thus, the notched impact strength monotonically increased.

For neat 100S, the fracture surface is flat (Figure 4c), which is thought to be a macroscopically brittle-like surface. However, at high magnification, elongated fibrils can be found throughout the fracture surface. The occurrence of fibrillation was attributed to the high stretching and shrinkage of the molecular chain segments under the impact load, which also consumed a large amount of energy during the crack propagation. Thus, the notched impact strength of 100S was much larger than that of 8008. C−100S−2.8% (Figure 4d) showed a rougher fracture surface than neat 100S with fiber pull-out, which introduced new energy consumption into the material. However, at high magnification, the fibrillation disappeared. Moreover, due to the poor wettability between the fibers and 100S, the fibers were “lying” parallel to the fracture surface, which increased the stress concentration of the surrounding matrix and caused brittle failure even at low stress. Thus, the energy consumed by plastic deformation was largely reduced. The notched impact strength of C−100S was dependent on the increased energy consumption by the fiber pull-out and the decreased energy consumed by the matrix plastic deformation. For C−100S−2.8%, the increased energy consumption by the fiber pull-out played a decisive role. Thus, the impact strength increased. With an increasing fiber volume fraction, the decreased energy consumed by the matrix plastic deformation played a decisive role. Thus, the impact strength decreased.

Neat K8303 (Figure 4e) and C4220 (Figure 4g) showed macroscopically ductile characteristics, as large-sized stretched dimples were observed covering the fracture surfaces. The appearance of the dimples can be attributed to the higher ethylene content and higher molecular weights of K8303 and C4220, and it also consumed a large amount of energy [29]. Thus, K8303 and C4220 showed much higher impact strengths than 8008 and 100S.

C−K8303−2.8% (Figure 4f) showed obvious macroscopic fluctuations, with many fibers pulled out. However, the high magnification micrograph provided a completely different view of the fracture surface than the low magnification. The matrix fractures were characterized by a much more brittle behavior, with cleavage fractures instead of dimples. As the notched impact strength of C−K8303−2.8% decreased by 54%, which indicated that the extra energy consumed by the fiber pull-out cannot compensate the reduced plastic energy. As the fiber volume fraction increased, more fibers were pulled out and more energy was consumed by the fiber pull-out, while the energy consumed by the plastic deformation was reduced, and these two mechanisms reached a balance. Thus, the notched impact strength for C−K8303 almost maintained a constant.

C−C4220−2.8% (Figure 4h) also resembled a macroscopically ductile-like surface involving fiber pull-out. However, at high magnification, it showed a brittle surface with less plastic deformation of the polymers. Thus, the notched impact strength of C−C4220−2.8% sharply decreased. As the fiber volume fraction increased, the extra energy consumed by the fiber pull-out was larger than that of the reduced energy by the polymer plasticity. Thus, the notched impact strength of C−C4220 increased slightly.

It can be concluded that the fibers introduced new energy consumption mechanisms in the LFT composites, but led to a more brittle fracture of the matrix, the energy consumed by which was largely decreased. Whether the impact strength increases or decreases with the addition of fibers depends on which mechanism predominates. Generally, for brittle polymers, new mechanisms introduced into composites play a crucial role in composite fracture. Thus, the toughness increases [38]. For ductile polymers, a decreased matrix plastic energy plays a crucial role in the fracture of the composite. Thus, the toughness decreases.

## 5. Prediction of Fracture Mode

Based on the investigations above, it can be seen that the physical and mechanical properties of the matrix influence the tensile and impact strengths of the LFT composites by four factors: the MFI, crystallinity, polymer tensile modulus, and polymer notched impact strength. The polymer properties simultaneously play a determined role in the fracture mode of LFT composites. Previous studies [39,40] have demonstrated that as cracks are initiated at the fiber ends, cracks propagate in the matrix or along the fiber−matrix interface, determined by the interfacial shear strength and the matrix shear strength. If the interfacial shear strength is larger than the matrix shear strength, the damage will propagate in the matrix and vice versa. Thus, it is important to know the interfacial shear strength to determine the realistic fracture mode in an LFT composite.

Generally, the interfacial shear strength, *τ*, can be obtained by micro-mechanical tests [41]. However, owing to the large standard deviation of micro-tests in actual experiments, it is difficult to obtain the exact value of *τ* directly.

Researchers have proposed an effective method based on the Kelly−Tyson model and the Bowyer−Bader model to obtain the value of *τ*. The details can be found in previous work [42]. In this study, the *τ* was obtained by this process and the results are presented in Table 4.

The matrix shear strength, *τ_m_*, can be obtained using $\tau_{m}=\sigma_{m}/\sqrt{3}$ according to the Von Mises yield criterion [43,44], where *σ_m_* is the tensile strength of the matrix. The values of *τ_m_* are also summarized in Table 4. Then, the fracture mode can be predicted by comparing *τ* and *τ_m_*. If *τ* > *τ_m_*, a matrix fracture occurs prior to interface debonding. Otherwise, interface debonding occurs prior to a matrix fracture [39,40].

For C-8008-2.8%, *τ* < *τ_m_*, and, theoretically, interfacial debonding occurs before the resin matrix failure. However, due to the small difference between the two values of *τ* and *τ_m_*, and the fact that defects such as pores existed in the polymer, it is also possible that the polymer failure occurs before the interface debonding. That means that both interface debonding and polymer fracture occurred in the C-8008-2.8% composites. This prediction can be proved by Figure 4b, in which both fiber pull-out and a matrix brittle fracture can be found together.

For C−100S−2.8%, the value of *τ* is much smaller than *τ_m_*. Thus, interfacial debonding occurred prior to polymer fracture. In Figure 4d, it can be seen that a large number of fibers were pulled out from the polymer, with a smooth fiber surface, indicating that cracks propagated along the interface. Moreover, there is still obvious plastic deformation of the polymer; however, this is greatly reduced compared with that of the neat polymer, indicating that the load of the resin is far from its limit load.

For C−K8303−2.8%, the value of *τ* is much larger than *τ_m_*. Thus, the polymer fracture is the determining fracture mode. In Figure 4f, the resin matrix shows typical brittle fracture characteristics with a cleavage structure, indicating that the stress concentration generated by the surrounding fibers was much greater than the bearing capacity of the resin. The polymer fractured with a much more brittle-like mode, consuming less energy. The remaining energy further diffused and finally caused the interface debonding and fiber pull-out.

For C−C4220−2.8%, it is a similar situation as that of C−K8303−2.8%. In Figure 4h, it can be seen that the polymer fractured with a much more brittle mode, with fiber pull-out and interface shear debonding.

Thus, for C−100S−2.8% and C−8008−2.8%, an increasing interfacial shear strength can improve the LFT mechanical properties. However, due to the similar values of *τ* and *τ_m_* in C−8008−2.8%, solely increasing the value of *τ* will not show an obvious modification. For C−K8303−2.8% and C−C4220−2.8%, since the polymer fracture determined the composite’s fracture failure, increasing the interface bonding strength had little effect on the composite’s strength.

## 6. Conclusions

In this study, the influence of the polymer matrices on the tensile and impact properties of the LFT composites was investigated, and the fracture modes for the LFT composites were predicted. The results showed that since neat 8008 showed the highest MFI and crystallinity, the fibers were more uniformly distributed in the matrix, and stress was transferred from the matrix to the fibers more efficiently. The corresponding LFT composites showed the highest tensile strength compared to the other three composites with the same fiber volume fractions. The polymer modulus played an important role in the LFT modulus. Since neat 8008 and neat K8303 had a higher tensile modulus than C4220 and 100S, C−8008 and C−K8303 showed a much higher tensile modulus than C−C4220 and C−100S. The toughness of the polymer played a vital role in determining whether the polymer can be toughened by the fibers. For brittle polymers, new mechanisms introduced into composites played a crucial role in the fracture of the composite. Thus, the toughness increased. For the ductile polymers, the decreased matrix plastic energy played a crucial role in the fracture of the composite. Thus, the toughness decreased.

Moreover, the interfacial shear strength of the LFT composites was calculated and the fracture modes were predicted. For C−100S−2.8% and C−8008−2.8%, which fractured with interfacial debonding, increasing the interfacial shear strength can improve the LFT mechanical properties. For C−K8303−2.8% and C−C4220−2.8%, which fractured with matrix shear failure, increasing the interface bonding strength would have little effect on the composites’ mechanical properties.

## Figures and Tables

**Table 1:** Basic physical and mechanical properties of polymer matrices [24,25,26,27].

Matrices	Number Average Molecular Weight (Mn)	Weight Average Molecular Weight (Mw)	Molecular Weight Distribution Coefficient
8008	17,200	72,400	4.21
100S	17,732	268,597	15.15
C4220	134,521	547,694	4.07
K8303	56,500	242,100	4.30

**Table 2:** MFI values of the neat matrices and the corresponding LFT composites.

Matrices	8008	100S	C4220	K8303
MFI g/10 min	5.19	0.05	0.10	0.90
LFT	B−8008−10.6%	B−100S−10.6%	B−C4220−10.6%	B−K8303−10.6%
MFI g/10 min	3.92	0.014	-	-

**Table 3:** The crystallinities of the neat matrices and the corresponding LFT composites.

Matrices	8008	100S	K8303	C4220
Crystallinity %	69.18	57.61	27.16	15.21
LFT	C−8008−2.8%	C−100S−2.8%	C−K8303−2.8%	C−C4220−2.8%
Crystallinity%	62.84	53.43	26.71	14.07

**Table 4:** Calculated *τ* and *τ_m_* for the composites and corresponding fracture mechanisms.

Composites	C−8008−2.8%	C−100S−2.8%	C−K8303−2.8%	C−C4220−2.8%
*τ*/MPa	9.8	6.4	18.6	14.1
*τ_m_*/MPa	10.32	12.61	10.20	11.46
fracture mode	D/M	D	M	M

## Data Availability

Data are available upon request due to restrictions, e.g., privacy or ethical reasons.
